# Genetic Determinants of Serum Calcification Propensity and Cardiovascular Outcomes in the General Population

**DOI:** 10.3389/fcvm.2021.809717

**Published:** 2022-01-14

**Authors:** Amber de Haan, Fariba Ahmadizar, Peter J. van der Most, Chris H. L. Thio, Zoha Kamali, Alireza Ani, Mohsen Ghanbari, Layal Chaker, Joyce van Meurs, M. Kamran Ikram, Harry van Goor, Stephan J. L. Bakker, Pim van der Harst, Harold Snieder, Maryam Kavousi, Andreas Pasch, Mark Eijgelsheim, Martin H. de Borst

**Affiliations:** ^1^Division of Nephrology, Department of Internal Medicine, University of Groningen, University Medical Center Groningen, Groningen, Netherlands; ^2^Department of Epidemiology, Erasmus Medical Center, University Medical Center Rotterdam, Rotterdam, Netherlands; ^3^Julias Global Health, University Medical Center Utrecht, Utrecht, Netherlands; ^4^Department of Epidemiology, University of Groningen, University Medical Center Groningen, Groningen, Netherlands; ^5^Department of Bioinformatics, Isfahan University of Medical Sciences, Isfahan, Iran; ^6^Division of Endocrinology, Department of Internal Medicine, Erasmus Medical Center, University Medical Center Rotterdam, Rotterdam, Netherlands; ^7^Department of Internal Medicine, Erasmus Medical Center, University Medical Center Rotterdam, Rotterdam, Netherlands; ^8^Department of Neurology, Erasmus Medical Center, University Medical Center Rotterdam, Rotterdam, Netherlands; ^9^Department of Pathology and Medical Biology, University of Groningen, University Medical Center Groningen, Groningen, Netherlands; ^10^Department of Cardiology, University Medical Center Utrecht, Utrecht, Netherlands; ^11^Calciscon AG, Biel, Switzerland; ^12^Institute for Physiology and Pathophysiology, Johannes Kepler University Linz, Linz, Austria

**Keywords:** calcification propensity, serum T_50_, GWAS, cardiovascular disease, population genetics, AHSG, fetuin-A

## Abstract

**Background:**

Serum calciprotein particle maturation time (T_50_), a measure of vascular calcification propensity, is associated with cardiovascular morbidity and mortality. We aimed to identify genetic loci associated with serum T_50_ and study their association with cardiovascular disease and mortality.

**Methods:**

We performed a genome-wide association study of serum T_50_ in 2,739 individuals of European descent participating in the Prevention of REnal and Vascular ENd-stage Disease (PREVEND) study, followed by a two-sample Mendelian randomization (MR) study to examine causal effects of T_50_ on cardiovascular outcomes. Finally, we examined associations between T_50_ loci and cardiovascular outcomes in 8,566 community-dwelling participants in the Rotterdam study.

**Results:**

We identified three independent genome-wide significant single nucleotide polymorphism (SNPs) in the *AHSG* gene encoding fetuin-A: rs4917 (*p* = 1.72 × 10^−101^), rs2077119 (*p* = 3.34 × 10^−18^), and rs9870756 (*p* = 3.10 × 10^−8^), together explaining 18.3% of variation in serum T_50_. MR did not demonstrate a causal effect of T_50_ on cardiovascular outcomes in the general population. Patient-level analyses revealed that the minor allele of rs9870756, which explained 9.1% of variation in T_50_, was associated with a primary composite endpoint of all-cause mortality or cardiovascular disease [odds ratio (95% CI) 1.14 (1.01–1.28)] and all-cause mortality alone [1.14 (1.00–1.31)]. The other variants were not associated with clinical outcomes. In patients with type 2 diabetes or chronic kidney disease, the association between rs9870756 and the primary composite endpoint was stronger [OR 1.40 (1.06–1.84), relative excess risk due to interaction 0.54 (0.01–1.08)].

**Conclusions:**

We identified three SNPs in the *AHSG* gene that explained 18.3% of variability in serum T_50_ levels. Only one SNP was associated with cardiovascular outcomes, particularly in individuals with type 2 diabetes or chronic kidney disease.

## Introduction

Arterial calcification is associated with an increased risk of cardiovascular morbidity and mortality in the general population, independent of traditional cardiovascular risk factors such as hypertension, smoking, and dyslipidemia (1–3). Patients with type 2 diabetes (T2D) or chronic kidney disease (CKD) are more prone to develop arterial calcification, and associations with cardiovascular outcomes are stronger in these high-risk populations (4, 5).

Calcification propensity can be quantified in serum using the T_50_-test, which evaluates the transformation time from primary to secondary calciprotein particles (CPPs) in supersaturating conditions of calcium and phosphate (6). Calcium and phosphate precipitation in serum is prevented by the formation of primary CPPs. Primary CPPs can spontaneously transform into secondary CPPs, which can induce calcification in vascular smooth muscle cells (7). The transformation time (T_50_) depends on the interplay and balance between calcification promoters (e.g., calcium, phosphate) and inhibitors (e.g., fetuin-A, magnesium).

A high calcification propensity, denoted by a low T_50_ value, has been associated with higher CT-based coronary calcification scores (8) and an increased risk of cardiovascular events (9) and all-cause mortality in CKD patients (10, 11), hemodialysis patients (9, 12), and kidney transplant recipients (13, 14). We recently reported an association between a lower T_50_ and a higher risk of cardiovascular mortality in the general population, which was more pronounced in patients with T2D at baseline (15).

The inter-individual variance in both arterial calcification and serum calcification propensity is likely in part determined by genetic factors (12, 16). Genome-wide association studies (GWASs) have identified multiple loci associated with arterial calcification (17), while no studies have so far reported genetic loci linked with calcification propensity.

In this study, we hypothesized that genetic factors driving serum T_50_ are linked with an increased risk of adverse cardiovascular outcomes, particularly in patients at high cardiovascular risk. We first identified genetic variants that were genome-wide associated with serum T_50_. Subsequently, we studied the association between serum T_50_ loci and cardiovascular outcomes in the general population and high-risk populations with T2D and/or CKD.

## Materials and Methods

### Study Design and Populations

A flowchart summarizing the study design and numbers of individuals available for each analysis is provided in Supplementary Figure 1. We had access to two cohort with GWAS data: the Prevention of REnal and Vascular ENd-stage Disease (PREVEND) study and the Rotterdam Study. Since T_50_ data were only available in the PREVEND study (and the Rotterdam Study had a larger sample size), we adopted a two-stage approach. First, we performed a GWAS in the PREVEND study (18) to identify single nucleotide polymorphisms (SNPs) associated with serum T_50_. Second, we examined the association between T_50_ SNPs, derived from the PREVEND GWAS, and cardiovascular outcomes in the Rotterdam Study (19). Finally, we performed a two-sample Mendelian randomization (MR) to examine potential causal effects of T_50_ on a range of cardiovascular outcomes. A brief description of each study is provided in the Supplementary Methods. Both PREVEND and the Rotterdam Study have been approved by local ethics committees and were conducted in accordance with the Declaration of Helsinki. Written informed consent was obtained from all study participants.

### Measurements and Definitions

Serum T_50_ measurements were performed in PREVEND as described previously (14). The analytical coefficients of variation of standards precipitating at 120, 260, and 390 min were 7.8, 5.1, and 5.9%, respectively. In PREVEND, serum T_50_ follows a normal distribution as reported previously (15).

The estimated glomerular filtration rate (eGFR) was calculated using the CKD-EPI equation, taking into account age, sex, and race (20). CKD was defined as an eGFR <60 ml/min/1.73m^2^. T2D was defined as fasting plasma glucose ≥7.0 mmol/L, random plasma glucose ≥11.1 mmol/L, or use of glucose-lowering medication. Hypercholesterolemia was defined as total serum cholesterol >6.5 mmol/L or a serum cholesterol >5 mmol/L if a history of myocardial infarction was present, or usage of lipid-lowering medications.

In the present study, smoking was defined as current smoking (including participants who had quit smoking <1 year before baseline) or non-smoking (including participants who had quit smoking at least 1 year before baseline). Hypertension was defined as systolic blood pressure (SBP) >140 mmHg, diastolic blood pressure (DBP)>90 mmHg, or self-reported use of blood pressure-lowering medications.

### Genotyping

Genotyping in PREVEND was performed using the Illumina Human CytoSNP-12 v2 chip. Samples were excluded in case of call rate <95%, duplication, or sex inconsistencies between database and genotypes. We also excluded SNPs with a call rate <95%, a minor allele frequency <1%, or a deviation from Hardy-Weinberg equilibrium (*P*-value <1 × 10^−5^). Principal component analysis was used to assess population stratification; samples with a *Z*-score >3 for the first five principal components were excluded. Variants were imputed using 1,000 Genomes, phase 1 version 3, as reference panel (21) using Minimac software (22). Designated risk alleles were those associated with lower serum T_50_ levels. Genotypes were represented as continuous allelic dosages from 0 to 2, reflecting an additive model.

In the Rotterdam Study, genotyping was performed using the Infinium II HumanHap550K Genotyping BeadChip version 3 (Illumina, San Diego, California, USA). SNPs that were independently associated with serum T_50_ were extracted from GWAS data.

### GWAS Analysis

GWAS was performed in PREVEND participants using linear regression with an additive SNP model adjusting for age, age^2^, sex, and the first 10 principal components to adjust for potential population stratification using SNPtest. A threshold of *P* < 5 × 10^−8^ was considered to represent genome-wide significance. Conditional analysis using the dosage of the most significant SNP as a covariate was performed in PLINK 1.9 to discern any secondary hits with independent effects. Upon discovering an independent secondary hit, the conditional analysis was repeated using both the top hit and the top secondary hit to see if there were further independent hits. This process was repeated until there were no more genome-wide significant hits. LocusZoom plots for each independent genome-wide significant hit were created using LocusZoom software (23). To determine the percentage of explained variance (*R*^2^) by the identified SNPs, we report the difference in *R*^2^ models (including age and sex) with and without the SNPs.

### Bioinformatics Characterization of T_50_ SNPs

We conducted several post-GWAS investigations to gain more knowledge about possible effects of the identified T_50_ SNPs. Firstly, we used an *in silico* sequencing pipeline (24) to investigate the functional characteristics of the selected SNPs and their vicinity (1Mb at either side). SNPs in moderate to high linkage disequilibrium (LD) (*r*^2^ > 0.5) with our index SNPs were selected for further analysis. Tabix (21), VCFtools (22), PLINK (25), and ANNOVAR (26) software packages were used for generating the appropriate variant call format files, SNP filtering, LD calculation, and variant annotation, respectively. Finally, the GWAS Catalog (27) database (e100, r2020-07-14) was used to identify the associated trait or outcome with each variant. All reports are based on available data from the 1,000 Genomes Project (GRCh37, Phase 3).

Secondly, we examined potential regulatory effects on gene expression (expression quantitative trait loci, eQTL) and splicing (splicing quantitative trait loci, sQTL) for the identified SNPs in liver tissue using the Genotype-Tissue Expression database (GTEx v8) (28). In addition, we used the Phenoscanner database (29) to determine any regulatory effects at the protein level (protein quantitative trait loci, pQTL). Finally, we performed two types of pathway analyses to identify potentially involved mechanisms. We used the GeneNetwork algorithm (30) based on co-regulation data and the GeneMANIA algorithm (31) based on composite networks of different data types. For the latter, we used the default setting, in which the algorithm expands the input query gene to the top-20 relevant genes for functional enrichment analysis.

### Mendelian Randomization

We applied two-sample MR methods to examine potential causal effects of T_50_ on a range of cardiovascular outcomes: coronary artery disease, coronary atherosclerosis, major coronary heart disease event, heart failure, and stroke, as well as subtypes of stroke. Before performing the MR, we verified the following key assumptions: relevance (SNPs are strongly related to the exposure), independence (SNPs are not associated with any confounder in the exposure-outcome relation), and exclusion restriction (SNPs affect the outcome exclusively through the exposure) (32). The (GWAS-derived) lead SNP was used as an instrument for T_50_. This SNP was extracted from publicly available GWAS data on outcomes from the IEU Open GWAS project repository (https://gwas.mrcieu.ac.uk/). We estimated causal effects of T_50_ on outcomes by calculating the Wald ratio, i.e., the lead SNP-outcome effect (in log-odds) divided by the lead SNP-T_50_ effect. Standard errors for the Wald ratio were approximated by the delta method using the *TwoSampleMR* (33) R package.

Additionally, we performed MR using all lead SNPs for T_50_. Here, a generalized weighted linear regression model (34) was used to obtain inverse variance weighted and MR Egger (35) pooled causal estimates of single SNP Wald ratios, while allowing for correlation (LD *r*^2^ according to the 1,000 G EUR reference population) between the lead SNPs, using the *MendelianRandomization* (34) R-package. We examined Cochran's Q statistic and Egger intercept to identify bias due to potential pleiotropy.

### Patient-Level Outcome Analyses

We analyzed patient-level outcomes in the Rotterdam Study given the larger sample size and the larger numbers of patients with T2D and CKD in this cohort, compared with the PREVEND cohort. The primary outcome was defined as a composite endpoint compromised of all-cause mortality and cardiovascular disease (CVD) as defined below. Secondary endpoints were isolated all-cause mortality, cardiovascular mortality, CVD, coronary heart disease (CHD), stroke, and heart failure (HF). In the Rotterdam Study, CVD was defined as the presence of CHD, stroke, or HF. The definitions and procedures on the adjudication of CVD outcomes have been described previously (36, 37). CHD is defined as fatal or non-fatal myocardial infarction, surgical or percutaneous coronary revascularization procedure, or death from CHD. Stroke was defined according to World Health Organization criteria with symptoms lasting 24 h or longer, which leads to death, with no apparent origin other than vascular. HF was defined according to the European Society of Cardiology guidelines as the combination of typical symptoms and signs confirmed by objective evidence of cardiac dysfunction or a positive response to initiated treatment (38).

### Statistical Analyses

We used logistic regression analysis to calculate odds ratios (ORs) and 95% confidence intervals (CIs) for the association between each T_50_ variant and clinical endpoints. We performed logistic regression analysis in the full cohort of the Rotterdam Study. We subsequently defined four subgroups based on vascular risk: (1) high-risk subgroup of participants with T2D, CKD or both, (2) subgroup with T2D only, (3) subgroup with CKD only, and (4) low-risk subgroup of participants without T2D or CKD. Logistic regression was used to cover both prevalent and incident cases of CVD, increasing statistical power. In high-risk subgroups, prevalent cases were only considered cases if a cardiovascular event happened after onset of T2D or CKD. We first calculated ORs adjusted for age and sex. In a second model, we additionally adjusted for hypertension, smoking status and hypercholesterolemia. SNPs were treated as continuous variables.

To evaluate potential existence of additive interaction between high-risk subgroup (dichotomous), SNPs (continuous), and outcomes (dichotomous), we calculated relative excess risk due to interaction (RERI). For calculation of RERI, we used the following formula: RERI = OR_A_ × OR_B_ × OR_AB_ – OR_A_ – OR_B_ + 1 (39), where OR_A_ refers to the effect of the SNP, OR_B_ to the effect of the high-risk group, and OR_AB_ is the effect of SNP × high-risk group. We obtained 95% CI using a tool provided by Knol et al. (40), which uses the delta method (41). If there was no additive interaction, the 95% CI of RERI would include 0 or RERI = 0.

Statistical analyses were performed using R Statistical software (version 4.03; R Foundation for Statistical Computing, Vienna, Austria) and IBM SPSS Statistics for Windows, version 23.0 (IBM Corporation, Armonk, NY, USA). A two-sided significance level of 0.05 was used unless stated otherwise. Skewed variables were log-transformed to achieve normal distribution. Continuous variables are reported as mean with standard deviation (SD) or as median with interquartile range (IQR) in case of skewed distributions. Categorical variables are presented as total numbers with corresponding percentages.

## Results

### Baseline Characteristics

Baseline characteristics of the cohorts are shown in Table 1. In PREVEND, mean age was 49.6 ± 11.9 years (mean ± SD), 48.1% were female, and mean BMI was 26 ± 4.1 kg/m^2^. The prevalence of T2D at baseline was 1.9% and the prevalence of CKD was 2.0%. At baseline, 31% of PREVEND participants had hypertension, 36.5% were smokers (or stopped smoking <1 year ago), and 26.1% had hypercholesterolemia.

**Table 1:** Baseline characteristics of the PREVEND cohort and the Rotterdam Study (full cohort and subgroups).

	**PREVEND**	**Rotterdam study**				
	**Full cohort**	**Full cohort**	**Low risk**	**High risk**	**T2D**	**CKD**
			**No T2D or CKD**	**T2D and/or CKD**		
*N*	2,739	8,566	6,584	1,774	1,075	833
Age (years), mean (SD)	49.6 (11.9)	65.1 (9.9)	63.5 (9.1)	71.2 (10.0)	68.4 (9.8)	75.5 (8.7)
Females, *n* (%)	1,317 (48.1)	4,838 (56.5)	3,786 (57.5)	935 (52.7)	513 (47.7)	489 (58.7)
BMI (kg/m^2^), mean (SD)	26.0 (4.1)	27.3 (4.2)	26.9 (4.0)	28.6 (4.6)	29.4 (4.8)	27.7 (4.1)
Current smoker, *n* (%)	1,000 (36.5)	1,614 (18.8)	1,302 (19.8)	273 (15.4)	180 (16.7)	109 (13.1)
Hypertension, *n* (%)	849 (31.0)	5,412 (63.2)	3,807 (57.8)	1,478 (83.3)	894 (83.2)	710 (85.2)
Hypercholesterolemia, *n* (%)	714 (26.1)	3,344 (39)	2,490 (37.8)	783 (44.1)	464 (43.2)	385 (46.2)
LDL cholesterol, mean (SD)	3.5 (0.97)	4.0 (0.96)	4.0 (0.95)	4.0 (1.0)	3.8 (0.98)	4.1 (1.0)
HDL cholesterol, mean (SD)	1.3 (0.40)	1.4 (0.41)	1.4 (0.41)	1.3 (0.36)	1.2 (0.35)	1.3 (0.4)
Total cholesterol, mean (SD)	5.6 (1.1)	5.7 (1.0)	5.8 (1.0)	5.6 (1.1)	5.4 (1.1)	5.7 (1.1)
Triglycerides, median (IQR)	1.2 (0.83–1.7)	1.3 (1.0–1.8)	1.3 (0.97–1.6)	1.6 (1.2–2.1)	1.7 (1.2–2.3)	1.5 (1.2–2.1)
T2D, *n* (%)	53 (1.9)	1,075 (12.5)	-	1,075 (60.5)	1,075 (100)	134 (16.1)
CKD, *n* (%)	56 (2.0)	833 (9.7)	-	833 (47.0)	134 (12.5)	833 (100)

*Baseline characteristics of the two cohorts, including subgroups of the Rotterdam Study. Low-risk subgroup: individuals free from type 2 diabetes and chronic kidney disease. High-risk subgroup: individuals with type 2 diabetes, chronic kidney disease, or both. Data are presented as mean (SD), median (interquartile range), or percentage*.

*BMI, Body mass index; CKD, chronic kidney disease; SD, standard deviation; T2D, type 2 diabetes*.

In the Rotterdam Study, mean age was 65.1 ± 9.9 years, 56.5% were female, and mean BMI was 27.3 ± 4.2 kg/m^2^. At baseline, 18.8% were smokers, 63.2% had hypertension, and 39% had hypercholesterolemia. The prevalence of T2D and CKD was 12.5% and 9.7%, respectively. Individuals in the high-risk (T2D and/or CKD), T2D, and CKD subgroups were older, more likely to have hypertension and hypercholesterolemia, and less likely to smoke than the full cohort and low-risk subgroup.

### GWAS

**Figure 1:**
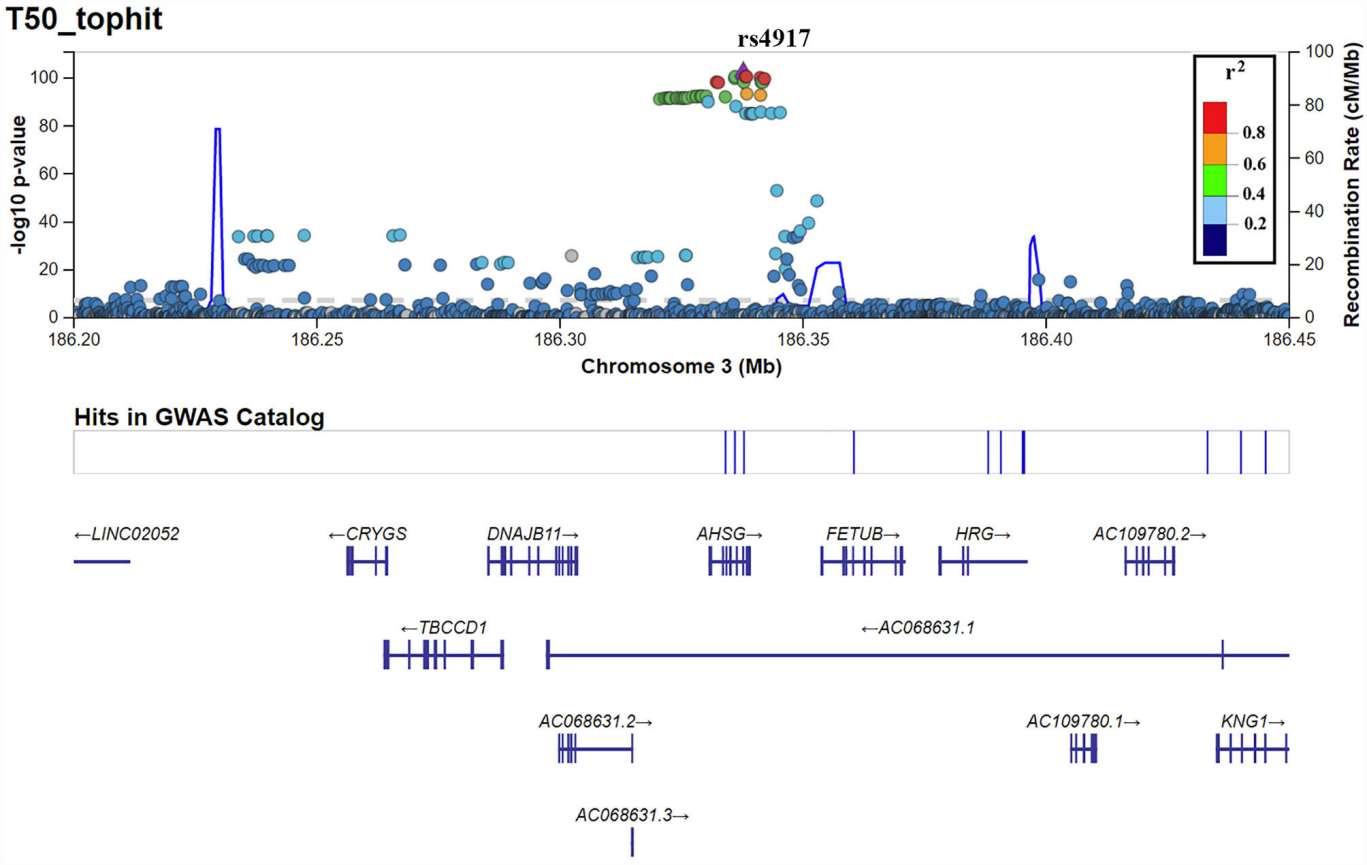
LocusZoom plot of the top hit, rs4917, from the T_50_ GWAS.

**Figure 2:**
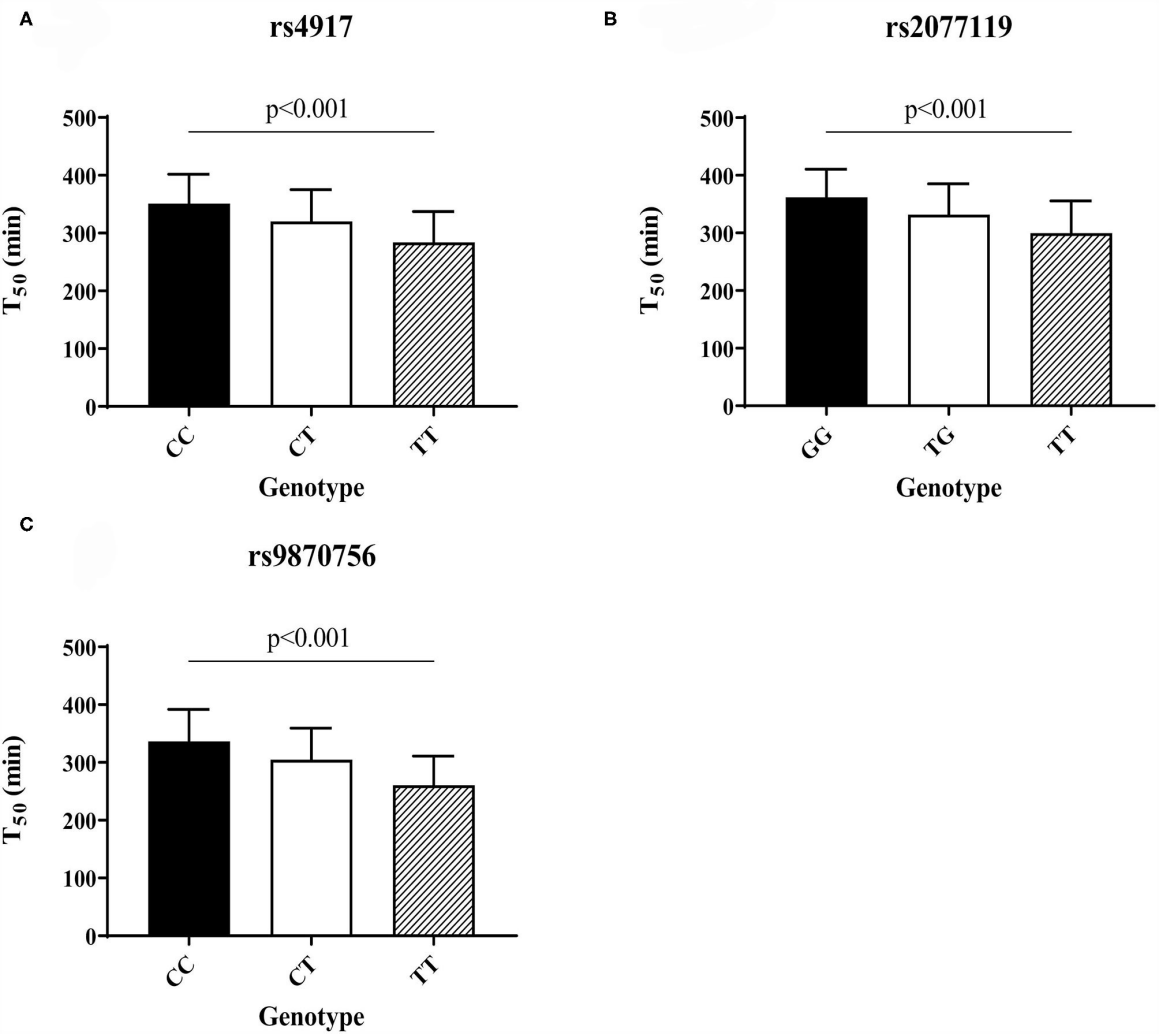
Serum T_50_ values for the different genotypes of the three independent T_50_ SNPs in the PREVEND cohort: **(A)** rs4917, **(B)** rs2077119, **(C)** rs9870756. Differences between genotypes calculated with ANOVA and *post-hoc* testing.

T_50_ GWAS in 2,739 PREVEND participants revealed 171 genetic variants in a single locus on chromosome 3 that were genome-wide significantly associated with serum T_50_ (Supplementary Table 1). The lead SNP was rs4917 (*p* = 1.27 × 10^−101)^), a missense variant in the *AHSG* gene encoding fetuin-A (also known as Alpha-2-HS-glycoprotein) (Figure 1). Carriers of the rs4917 minor T allele displayed lower serum T_50_ values (Figure 2A). Subsequent conditional analyses revealed two additional independent effects at rs2077119 (*p* = 3.34 × 10^−18^) and rs9870756 (*p* = 3.10 × 10^−8^) (Supplementary Figure 2, Supplementary Tables 2, 3). Individuals carrying the rs2077119 major T allele or the rs9870756 minor T allele had on average lower T_50_ values (Figures 2B,C). All three independent genome-wide significant SNPs are located at or nearby *AHSG*. Detailed information on the three SNPs is provided in Table 2. After correcting for age and sex, the lead SNP rs4917 explained 15% of variance in serum T_50_, while all three SNPs combined explained 18.3% of serum T_50_ variance (Supplementary Table 4).

**Table 2:** Details of three independent SNPs identified by T_50_ GWAS.

	**rsID**	**Chr:position (GRCh37)**	**Gene**	**Coded AF**		**Coded allele**	**Non-coded allele**	**Effect**	***P*-value**
				**PREVEND**	**RS**				
Top hit	rs4917	3:186337713	AHSG	0.381	0.358	T	C	−32.85	1.72e-101
After conditioning on rs4917	rs2077119	3:186330462	AHSG	0.575	0.568	T	G	−18.17	3.34e-18
After conditioning on rs4917 and rs2077119	rs9870756	3:186344614	AHSG	0.150	0.137	T	C	−14.87	3.10e-08

*AF, allele frequency; Chr, chromosome; RS, Rotterdam Study*.

### Bioinformatics Characterization of T_50_ SNPs

We identified 40 SNPs in moderate (*r*^2^ >0.5) or high LD (*r*^2^ >0.8) with rs4917 (Supplementary Table 5). Some SNPs were associated with susceptibility to leanness or blood protein levels, but most SNPs in LD with rs4917 had no known associations. The association with blood protein levels recurred for SNPs in LD with rs2077119. We could not identify SNPs in LD with rs9870756. Since the liver is the predominant expression site for fetuin-A, we subsequently studied potential consequences of the genetic variants in liver tissue. eQTL analysis did not identify any significant associations in liver tissue for the three SNPs (Supplementary Table 6). However, the T allele of both rs4917 and rs2077119 were significantly associated with a decreased intron excision ratio of the *AHSG* gene in liver tissue (Supplementary Figure 3). In addition, pQTL analysis in blood showed that the T allele of all three SNPs is significantly associated with lower plasma levels of the protein product of the *AHSG* gene, i.e., fetuin-A (Supplementary Table 7). Functional investigations of this gene, alongside its top 20 related genes in GeneMANIA, revealed its likely involvement in bone mineralization and function (Supplementary Table 8). Furthermore, in functional predictions based on co-regulation data, the majority of the top results converged on the coagulation cascade (Supplementary Tables 9–12).

### Mendelian Randomization

We found no significant causal effects of T_50_ on any of the examined outcomes (coronary artery disease, coronary atherosclerosis, major coronary heart disease event, heart failure, stroke, and subtypes of stroke) (Supplementary Figure 4, Supplementary Table 13). A suggestive, positive causal effect of T_50_ on small-vessel stroke was found (IVW OR = 1.001 per unit T_50_, 95% CI: 1.000–1.002, *P* = 0.054), in the absence of significant heterogeneity [*Q*_(df)_ = 0.723, *P*_heterogeneity_ = 0.395] or suggestive directional pleiotropy (*P*_Egger intercept_ = 0.594) (Supplementary Figure 4, Supplementary Table 13). However, this was only true for the IVW analysis using multi-ancestry GWAS data on small-vessel stroke, and not corroborated by Wald ratio and MR Egger estimates or European ancestry-only GWAS data.

### Patient-Level Outcome Analyses

#### Primary Endpoint

We subsequently studied whether the three independent T_50_ SNPs were associated with cardiovascular outcomes in the Rotterdam Study. Results for the primary composite endpoint (all-cause mortality and CVD) are presented in Table 3. The risk allele of rs9870756, but not the other two SNPs, was associated with an increased risk of the primary composite endpoint [OR (95% CI): 1.14 (1.01–1.28)] in the full cohort. Similarly, the T allele of rs9870756, but not the other two SNPs, was associated with an increased risk of the primary endpoint in the high-risk [1.40 (1.06–1.84)] and T2D subgroups [1.44 (1.03–2.02)], while a non-significant trend was observed in the CKD subgroup [1.52 (0.98–2.36)]. There were no significant associations in the low-risk subgroup. Additional adjustment for hypertension, smoking, and hypercholesterolemia did not materially alter the associations (Supplementary Table 14).

**Table 3:** Associations of three lead T_50_ SNPs with clinical outcomes in the Rotterdam Study.

	**Full cohort**	**Subgroups**			
		**Low risk**	**High risk**	**T2D**	**CKD**
		**No T2D or CKD**	**T2D and/or CKD**		
N	8,566	6,584	1,774	1,075	833
**Primary composite endpoint**
Events, n (%)	2,693 (35.7%)	1,853 (30.8%)	764 (57.3%)	416 (50.2%)	417 (70.8%)
rs4917	0.99 (0.91, 1.08)	0.98 (0.88, 1.08)	0.92 (0.76, 1.12)	0.94 (0.73, 1.21)	0.86 (0.64, 1.16)
rs2077119	0.95 (0.88, 1.04)	0.96 (0.87, 1.06)	0.87 (0.72, 1.06)	0.92 (0.72, 1.18)	0.75 (0.56, 1.01)
rs9870756	1.14 (1.01, 1.28)^*^	1.06 (0.93, 1.21)	1.40 (1.06, 1.84)^*^	1.44 (1.03, 2.02)^*^	1.52 (0.98, 2.36)
**All-cause mortality**
Events, n (%)	2,002 (26.6%)	1,307 (21.7%)	942 (48.2%)	343 (41.1%)	359 (61.0%)
rs4917	0.98 (0.89, 1.08)	0.95 (0.85, 1.06)	0.97 (0.80, 1.18)	0.99 (0.76, 1.29)	0.93 (0.70, 1.24)
rs2077119	0.96 (0.88, 1.05)	0.97 (0.87, 1.08)	0.86 (0.71, 1.04)	0.88 (0.68, 1.14)	0.81 (0.61, 1.06)
rs9870756	1.14 (1.00, 1.30)^*^	1.03 (0.89, 1.20)	1.41 (1.07, 1.86)^*^	1.43 (1.00, 2.04)^*^	1.60 (1.05, 2.42)^*^
**CVD**
Events, n (%)	1,121 (14.9%)	779 (12.9%)	303 (23.7%)	175 (21.1%)	159 (27.0%)
rs4917	1.05 (0.95, 1.16)	1.04 (0.92, 1.16)	1.05 (0.87, 1.28)	1.16 (0.89, 1.50)	0.99 (0.75, 1.29)
rs2077119	1.01 (0.92, 1.11)	1.01 (0.91, 1.13)	1.02 (0.85, 1.23)	1.04 (0.80, 1.34)	0.99 (0.76, 1.28)
rs9870756	1.11 (0.97, 1.27)	1.02 (0.87, 1.20)	1.34 (1.03, 1.74)^*^	1.55 (1.10, 2.17)^*^	1.12 (0.77, 1.62)

*Estimates from logistic regression analyses in the Rotterdam Study adjusted for age and sex. Data are presented as OR (95% CI) per risk allele. OR (95% CI) in bold were statistically significant*.

* *P < 0.05. Primary composite endpoint: all-cause mortality or CVD*.

*CKD, chronic kidney disease; CVD, cardiovascular disease; T2D, type 2 diabetes*.

#### Secondary Endpoints

Analysis of individual components of the primary composite endpoint revealed significant associations between the high-risk allele of rs9870756 and all-cause mortality in the full cohort [1.14 (1.00–1.30)] and in high-risk [1.41 (1.07–1.86)], CKD [1.60 (1.05–2.42)], and T2D [1.43 (1.00–2.04)] subgroups (Table 3). Similar associations were found for CVD (Table 3). The T allele of rs9870756 was associated with stroke in the full cohort and high-risk and T2D subgroups (Supplementary Table 15). No other significant associations were observed. Additional adjustments did not substantially alter the associations (Supplementary Table 16).

#### Additive Interaction

We found significant additive interaction by vascular risk status (high-risk vs. low-risk) for the association between rs9870756 and the primary composite endpoint [RERI (95% CI): 0.54 (0.01–1.08)], all-cause mortality [RERI: 0.62 (0.02–1.22)] and CVD [RERI: 0.58 (0.02–0.86)] (Table 4). There was no additive interaction for the other outcomes or SNPs (Supplementary Table 17).

**Table 4:** Additive interaction between SNPs and high-risk group.

	**SNP**	**High-risk group**	**SNP × high-risk group**	**RERI (95% CI)**
**Primary composite endpoint**
rs4917	0.99	1.59	0.91	−0.15 (−0.49, 0.19)
rs2077119	0.96	1.74	0.87	−0.25 (−0.63, 0.13)
rs9870756	1.06	1.35	1.36	0.54 (0.01, 1.08)^*^
**All-cause mortality**
rs4917	0.97	1.69	0.97	−0.07 (−0.45, 0.29)
rs2077119	0.97	2.04	0.83	−0.37 (−0.82, 0.10)
rs9870756	1.02	1.5	1.4	0.62 (0.02, 1.22)^*^
**CVD**
rs4917	1.05	1.2	1.02	0.04 (−0.24, 0.30)
rs2077119	1.01	1.14	1.06	0.07 (−0.18, 0.31)
rs9870756	1.02	1.1	1.39	0.43 (0.02, 0.86)^*^

* *P < 0.05*.

*CVD, cardiovascular disease; RERI, relative excess risk due to interaction; SNP, single nucleotide polymorphism. Statistically significant RERI values are highlighted in bold*.

## Discussion

To our knowledge, the present study is the first genome-wide examination for genetic variants associated with serum T_50_. We identified three SNPs in the *AHSG* gene independently associated with serum T_50_ at a genome-wide significant level. Post-GWAS investigations show that all three SNPs are associated with fetuin-A blood concentrations. MR analysis based on large, mostly population-based, publicly available GWAS summary statistics could not demonstrate significant causal effects of T_50_ on cardiovascular outcomes. In patient-level analyses in an independent second cohort, we found that rs9870756, which explained 9.1% of variation in T_50_, was associated with the primary composite endpoints of CVD and all-cause mortality in the general population. These associations were more pronounced in high-risk subpopulations with T2D and/or CKD, but were not observed for the other two lead T_50_ SNPs in *AHSG* (rs4917 and rs2077119).

The three genome-wide significant SNPs are all located in the *AHSG* gene, which encodes for fetuin-A. Although fetuin-A was not measured in PREVEND or the Rotterdam Study, and therefore we could not directly examine the T_50_ SNPs in relationship with fetuin-A levels, all three T_50_ SNPs identified in the current study have previously been reported to be genome-wide significant in a GWAS of plasma fetuin-A (42). We found a relatively high explained variance (18.3%) by only the three lead SNPs, in line with previous studies showing that rs4917 (43, 44) and SNPs in high LD with rs2077119 (44) explain up to 21% of the variance in fetuin-A concentrations. Fetuin-A, a calcification inhibitor synthesized and secreted predominantly by the liver, is a known determinant of serum T_50_ levels (45). The high explained variance of the three SNPs on serum T_50_ might therefore be driven by a strong effect on fetuin-A concentrations. This is supported by our bioinformatics characterization, which shows that the variants we report all affect the concentration of circulating fetuin-A.

Our findings suggest that fetuin-A plays a pivotal role in serum calcification propensity. This is in agreement with other studies that show a relationship between low serum fetuin-A levels and arterial calcification (46). Similarly, the T allele of rs4917 has been associated with both lower fetuin-A levels (42, 43) and arterial calcification (47). Nevertheless, a study in 1,649 Caucasians with suspected or established coronary artery disease found no association between serum fetuin-A levels or rs4917 and arterial calcification (48). One study in patients with diabetic nephropathy even found that higher circulating fetuin-A levels were associated with higher, not lower, coronary artery calcification scores (49). As a potential explanation, arteries with high calcification may produce fetuin-A as a counter-regulatory mechanism (50). We also found that the *AHSG* gene is associated with the coagulation cascade (Supplementary Tables 9–12). This may be explained by the fact that fetuin-A is related to interleukin-6 (51), which in turn is associated with coagulation (52). Another possibility is that low fetuin-A levels leading to inflammation, higher secondary CPP levels, and increased precipitation and destabilization of atherosclerotic plaques (53).

MR analyses did not confirm a causal effect of serum T_50_ on cardiovascular outcomes. We only found a suggestive positive causal effect of T_50_ on small-vessel stroke, but this finding was not robust. In addition, we cannot confidently exclude that any effect was driven by horizontal pleiotropy: as we used three correlated SNPs from the same region, directional pleiotropy as assessed by the MR Egger intercept may have limited value due to correlated effects of these SNPs. The MR analyses used publicly available GWAS data that were mostly based on the general population. Patient-level outcome analyses in the Rotterdam cohort showed additive interaction by prevalent T2D and/or CKD for the association between one of the three lead SNPs and CVD outcomes. A possible next step would be to perform MR analyses in patient populations with T2D and/or CKD, although there are currently insufficient large cohorts with GWAS data and selection bias may adversely impact such analyses (54).

A meta-analysis of *AHSG* variants found that rs4917 was not associated with CHD risk (43). These findings are confirmed in our study since we found no associations between rs4917, the lead SNP in the GWAS, and the risk of CHD, CVD, or mortality. However, we found that the minor allele of the rs9870756 SNP was associated with the primary composite endpoint and all-cause mortality as an individual endpoint, both in the general population and in high-risk subgroups. Although the minor allele of this variant has been linked with lower fetuin-A levels in a GWAS (42), this variant has not been reported in other prior studies. In addition, we report an additive interaction of rs9870756 and T2D and/or CKD on the primary composite endpoint, all-cause mortality, and CVD. This is in line with a previous study showing that the association between serum T_50_ and risk of cardiovascular mortality was stronger in participants with T2D compared with the general population (15). At the same time, since rs9870756 was the top tertiary SNP and, although rs9870756 in itself explained 9.1% of variation in T_50_, the primary and secondary top SNPs were not associated with clinical outcomes, our findings regarding the potential impact of T_50_ on cardiovascular outcomes and mortality should be interpreted with caution. Future studies, preferably in vascular high-risk population, should confirm the association between rs9870756 and cardiovascular outcomes.

Strengths of this study include the fact that this is the first GWAS for serum T_50_, the biologically plausible genome-wide significant SNPs in the gene encoding fetuin-A that explained a high percentage of variation in T_50_, and the extension of our analyses to clinical outcomes. Limitations include the limited GWAS sample size, allowing only variants with large effect size to be found, the PREVEND study composition of entirely participants of European descent, and the reference panel used for imputation in PREVEND (1,000 genomes, phase 1 version 3).

In conclusion, we identified three independent genetic variants in or near the *AHSG* gene, encoding the endogenous calcification inhibitor fetuin-A, that were genome-wide significantly associated with serum T_50_, a marker of calcification propensity. Although MR did not demonstrate causal effects of T_50_ on CVD outcomes, patient-level analyses in an independent cohort suggest that one of the three lead SNPs, rs9870756, is associated with all-cause mortality and CVD, and that this risk is more pronounced in patients with T2D or CKD. Larger studies are needed to confirm that a genetic predisposition for increased calcification propensity drives cardiovascular risk in individuals with T2D and/or CKD.

## Data Availability Statement

The original contributions presented in the study are included in the article/Supplementary Materials, further inquiries can be directed to the corresponding author/s.

## Ethics Statement

The studies involving human participants were reviewed and approved by the Medical Ethics Review Board of the University Medical Center Groningen and the Medical Ethics Review Committee of the Erasmus MC. The patients/participants provided their written informed consent to participate in this study.

## Author Contributions

AH, FA, PM, ZK, AA, and CT performed the analyses. AH wrote the first draft of the manuscript. PM, ZK, AA, CT, FA, MG, LC, JM, MI, HG, SB, PH, HS, MK, AP, ME, and MB gave feedback and contributed to manuscript revision. All authors have read and approved the submitted version.

## Funding

This work was co-funded by the PPP Allowance made available by Health~Holland, Top Sector Life Sciences and Health to stimulate public-private partnerships (grant numbers RVO/6320 and IMAGEN/LSHM20009). The PREVEND Study was funded by the Dutch Kidney Foundation (Grant E.033). The Rotterdam Study was supported by the Erasmus MC University Medical Center and Erasmus University Rotterdam; the Netherlands Organization for Scientific Research (NWO); the Netherlands Organization for Health Research and Development (ZonMw); the Research Institute for Diseases in the Elderly (RIDE); the Netherlands Genomics Initiative (NGI); the Ministry of Education, Culture and Science; the Ministry of Health, Welfare and Sports; the European Commission (DG XII); and the Municipality of Rotterdam. None of the above funding sources or sponsors were involved in the conception, execution, or analysis and interpretation of the data, and had no role in drafting the manuscript.

## Conflict of Interest

AP is an inventor of the T_50_ test and an employee of Calciscon AG, Biel, Switzerland, which commercializes the T_50_ test and holds stock in this company. The remaining authors declare that the research was conducted in the absence of any commercial or financial relationships that could be construed as a potential conflict of interest.

## Publisher's Note

All claims expressed in this article are solely those of the authors and do not necessarily represent those of their affiliated organizations, or those of the publisher, the editors and the reviewers. Any product that may be evaluated in this article, or claim that may be made by its manufacturer, is not guaranteed or endorsed by the publisher.
