# Supplementary material for: Genetic Determinants of Serum Calcification Propensity and Cardiovascular Outcomes in the General Population
Source: Front Cardiovasc Med. 2022 Jan 14;8:809717. doi: 10.3389/fcvm.2021.809717 (PMC8795369; doi:10.3389/fcvm.2021.809717)
Supplement: Supplementary file 1 [file Data_Sheet_1.docx]

Supplementary Material

# Supplementary Data

## PREVEND

PREVEND is a prospective cohort study designed to investigate the impact of increased urinary albumin levels on renal and cardiovascular outcomes in the general population. Details of this study have been described elsewhere (18). In short, a total of 8,592 participants were recruited from the general population in Groningen, the Netherlands, between 1997 and 1998. After extensive examination at baseline, participants completed four follow-up examinations in 2003, 2006, 2008 and 2012. Data from 2,739 PREVEND participants with genetic and serum T_50_ data were available for T_50_ GWAS analysis (Figure S1).

## The Rotterdam Study

The Rotterdam Study was started to study the etiology and natural history of chronic diseases in mid-life and late-life among inhabitants of the Ommoord district in Rotterdam (19). Briefly, this ongoing prospective cohort started in 1990, and initially, all inhabitants >55 years were invited to participate (RS-I). The cohort was subsequently expanded in 2000 (RS-II) and again in 2005 (RS-III), with the inclusion of individuals >45 years. Individuals are invited for an interview and an extensive set of examinations every 3-4 years. This study included participants (n=8,556) with GWAS data and no prevalent CVD in participants with T2D and/or CKD, from the third examination of the first cohort (RS-I-3, 1997-1999) and the first examinations of the second (RS-II-1, 2000-2001) and the third (RS-III-1, 2006-2008) cohorts.

# Supplementary Figures and Tables

## Supplementary Tables

See excel file “Supplementary Tables” for tables S1-S17.

## Supplementary Figures


 **Supplementary Figure S1**. Study design. The GWAS was performed in the PREVEND study and the association of serum T_50­_ loci with clinical outcomes was examined in the Rotterdam Study. Participants in the Rotterdam study were excluded if there was no data available on cardiovascular outcomes, no follow-up data, or prevalent cardiovascular disease before onset T2D or CKD. CKD=chronic kidney disease; GWAS=genome-wide association study; T2D=type 2 diabetes; CVD=cardiovascular disease.

**A**


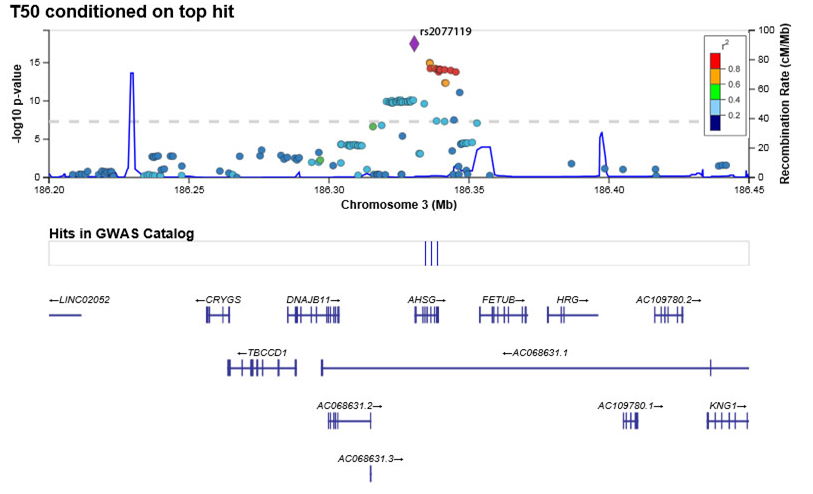

**B**
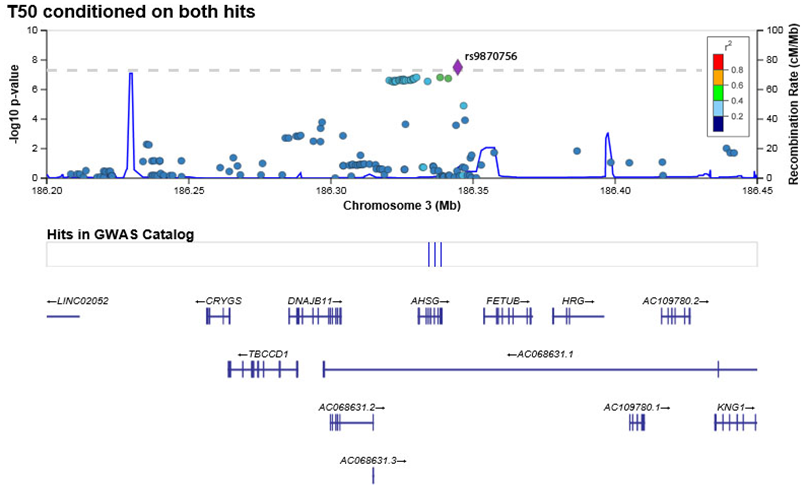
 **Supplementary Figure S2**. (**A**) Locuszoom plot of rs2077119, upon conditioning for rs4917. (**B**) LocusZoom plot of rs9870756, upon conditioning for rs4917 and rs2077119.

**A B**


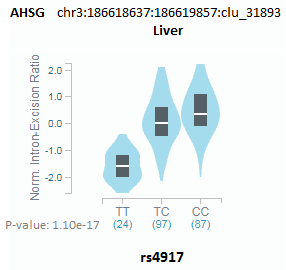

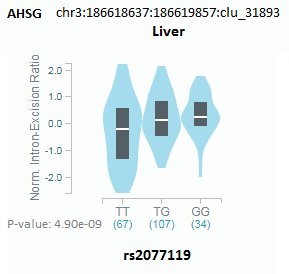


**Supplementary Figure S3**. Violin plots of splicing effects on *AHSG* intron expression in liver tissue for (**A**) rs4917 and (**B**) rs2077119.


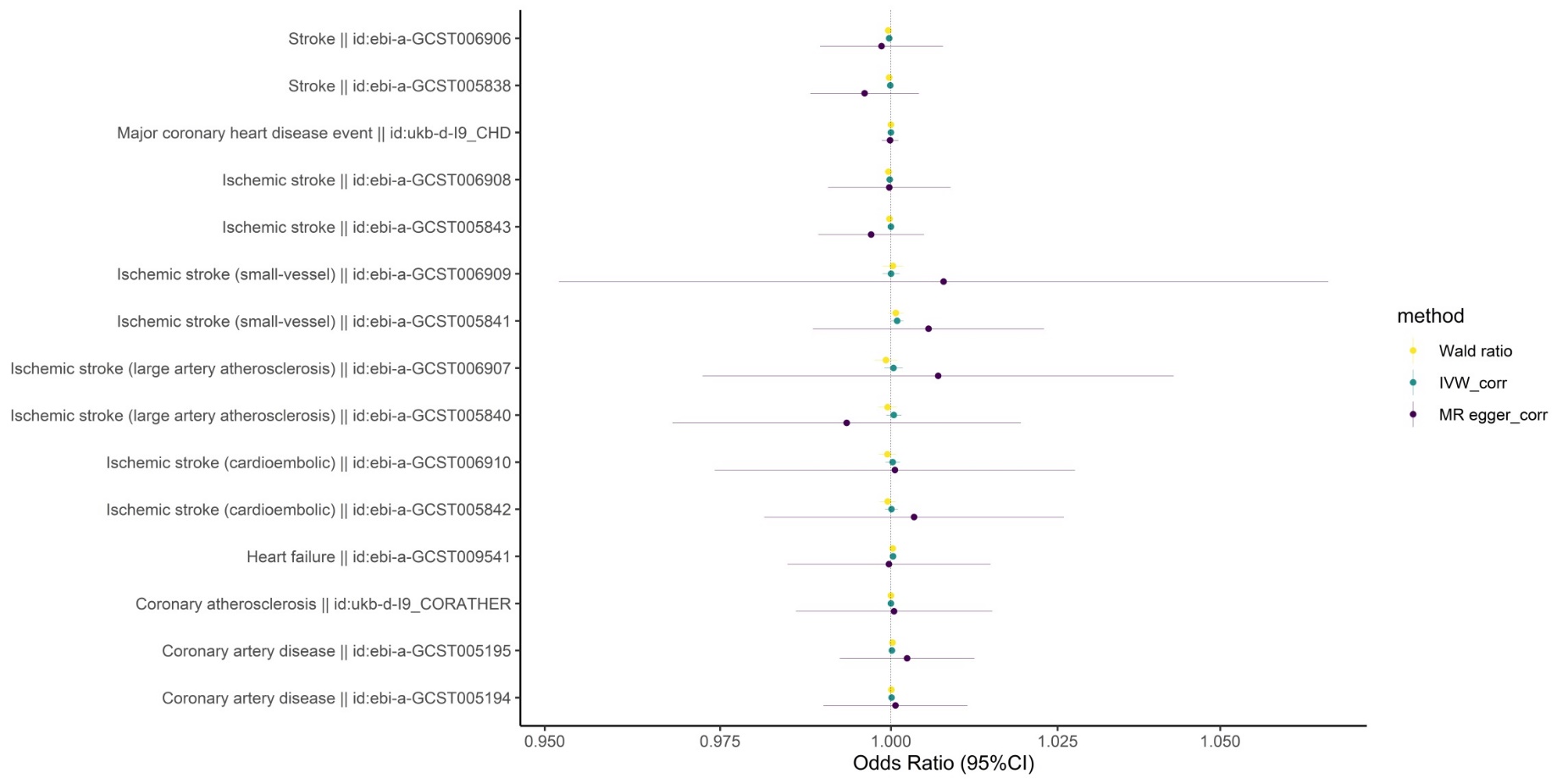
**Supplementary Figure S4.** Results from Mendelian randomization.
